# Study protocol: can a school gardening intervention improve children’s diets?

**DOI:** 10.1186/1471-2458-12-304

**Published:** 2012-04-26

**Authors:** Meaghan S Christian, Charlotte EL Evans, Mark Conner, Joan K Ransley, Janet E Cade

**Affiliations:** 1 Nutritional Epidemiology Group, School of Food Science and Nutrition, University of Leeds, Leeds, West Yorkshire, LS2 9JT, UK; 2 Institute of Psychological Sciences, University of Leeds, Leeds, West Yorkshire, LS2 9JT, UK

## Abstract

**Background:**

The current academic literature suggests there is a potential for using gardening as a tool to improve children’s fruit and vegetable intake. This study is two parallel randomised controlled trials (RCT) devised to evaluate the school gardening programme of the Royal Horticultural Society (RHS) Campaign for School Gardening, to determine if it has an effect on children’s fruit and vegetable intake.

**Method/Design:**

Trial One will consist of 26 schools; these schools will be randomised into two groups, one to receive the intensive intervention as “Partner Schools” and the other to receive the less intensive intervention as “Associate Schools”. Trial Two will consist of 32 schools; these schools will be randomised into either the less intensive intervention “Associate Schools” or a comparison group with delayed intervention. Baseline data collection will be collected using a 24-hour food diary (CADET) to collect data on dietary intake and a questionnaire exploring children’s knowledge and attitudes towards fruit and vegetables. A process measures questionnaire will be used to assess each school’s gardening activities.

**Discussion:**

The results from these trials will provide information on the impact of the RHS Campaign for School Gardening on children’s fruit and vegetable intake. The evaluation will provide valuable information for designing future research in primary school children’s diets and school based interventions.

**Trial registration:**

ISRCTN11396528

## Background

The impact of poor nutrition in children is causing major public health concerns across the globe [1]. Fruit and vegetables are a fundamental component of a healthy diet[2]. Currently, children’s consumption of fruit and vegetables is low in the United States of America, Australia and most European countries,[3-5] with the average intake of fruit and vegetables for children in the UK being around 2.5 servings per day[6]. In British children the main source of energy intake is from chips, biscuits and crisps,[6] highlighting the need for public health interventions to improve children’s overall dietary habits[7].

Epidemiological evidence indicates that a diet rich in fruit and vegetables can decrease the risk of developing cardiovascular disease, stroke, hypertension, type 2 diabetes mellitus, obesity and several forms of cancer[1,8]. A diet low in fruit and vegetable intake is one of the top ten risk factors for global mortality[9]. Research has also revealed that dietary habits are developed in childhood and persist throughout life; therefore it is vital that children at a young age consume adequate levels of fruit and vegetables[10,11]. Several studies indicate that children’s fruit and vegetable intake is positively associated with parental consumption[12].

Of particular public concern is the rise of obesity in children[13]. National surveys state that approximately one in ten children under the age of ten is obese, further estimates predict that 80% of children who are obese at the age of 10 to 14 will remain obese into adulthood[14]. Diet plays a fundamental role in weight management, having a healthy diet rich in fruit and vegetables, which are low energy density foods, could help tackle this epidemic[15].

Several different nutrition education programmes have been developed for schools, home and community settings in an attempt to improve children’s diets[15-22]. Evidence suggests that the most effective interventions are multi-component with both school and home based components [23,24]. Successful intervention studies have included a variety of components; integrating teaching about fruit and vegetables into the curriculum, training teachers in theories of behaviour change and nutritional education, increasing fruit and vegetable availability at school and in school meals, training of catering staff (verbal encouragement), hands-on exposure (tasting and preparation sessions), parental involvement through newsletters and homework activities, whole school approach (developing a nutrition policy, evening activities) and community involvement (local fruit and vegetable industry)[20,23-30]. These intervention programmes report a moderate increase in children’s fruit and vegetable consumption of approximately one third of a portion of fruit and or vegetables [22,31,32].

The use of school gardens as an education tool in schools is a relatively new approach to improve children’s diets. The theory behind using a school or community garden to improve children’s diets is that it provides children with the opportunity to learn about how fruit and vegetables are grown in an interactive manner[33], taking the focus away from classroom based nutrition education by using external support or a trained teacher.

The British Nutrition Foundation conducted a review of the psychosocial basis of food choice to provide evidence to explain how to influence food choice in children[34]. The main findings of relevance to young children were: the ‘one size fits all’ approach to intervention design does not seem to work well in any setting; and tailoring and message reinforcement appears to be important for sustained interest in the intervention programme. This is relevant from a school gardening perspective which requires repeated attention to prepare the ground, plant, tend and harvest. Several studies have indicated that the concept of familiarisation is important for children. Studies suggest that consumption of fruit and vegetables can be promoted if children are exposed to ‘healthier’ foods via teaching, through peer modelling, via the cafeteria and in vending machines. For example, children who were introduced to new foods using ‘hands-on’ activities in the classroom were 3–20 times more likely to subsequently choose and eat these foods in the canteen than children who did not have prior exposure[35-37].

This study will evaluate the Royal Horticultural Society (RHS) “Campaign for School Gardening.” This programme was launched in 2007 and since then has recruited over 11,500 primary schools. The main aims of the programme are to encourage schools to be involved in growing fruit and vegetables, to enrich the curriculum activities of the school, and to educate children with the values of gardening such as “healthy living” and “sustainability of the natural world [38].” This study will use two parallel randomised controlled trials to provide data on whether the RHS Campaign for School Gardening has an impact on fruit and vegetable intake in the diets of children. It will clarify the nature of any impact and provide important information on whether and how the children’s diets may be improved.

## Research questions

The following research questions apply to both Trials 1 and 2:

· Can the RHS Campaign lead to increases in vegetable and fruit intake in children aged 8–9 years?

· Does the RHS Campaign affect children's intake of other food and drink e.g. savoury snacks, confectionery products, soft drinks?

· What is the effect of the RHS Campaign on intake of key nutrients (fat, carbohydrate, protein, vitamin C, carotene, iron, sodium, folate)?

The effectiveness of either intervention would be determined by an increase in mean intake in one of the following; mean intake of fruit, mean intake of vegetables, or mean intake of fruit and vegetables at follow-up after adjusting for baseline.

## Methodology

### Sampling and recruitment of schools

#### Trial 1 – intensive vs. Less intensive intervention

The RHS plan to establish their Campaign for School Gardening to schools in the London region in the autumn of 2009. The RHS Campaign provides intensive support in each region to 10 schools through support from an RHS School Gardening Regional Advisor (the intensive intervention). The remaining schools will have access to support through twilight training sessions for staff and other activities (the less intensive intervention).

Twenty-six schools will be recruited from four boroughs in London: Wandsworth; Tower Hamlets; Greenwich; and Sutton. Of the 26 schools we will randomly allocate 10 schools to receive the intensive intervention and 16 schools to receive the less intensive intervention. The allocation sequence will be generated by the trial statistician. All schools will be allocated at the same time. Time between notification of allocation and the start of the intervention will be as short as possible. It will not be possible to randomise schools to receive no intervention at all since the RHS is committed to providing support to all schools who register an interest in the Campaign. As a consequence of this, we will recruit a second set of schools into a linked trial.

#### Trial 2 – less intensive vs. Delayed intervention

Following selection of schools into trial 1, we will contact schools from neighbouring boroughs in London: Lewisham; Lambeth; Merton and Newham. We anticipate that these boroughs will have approximately 130 primary schools. We will aim to recruit 32 schools into the second trial. Of these schools, 16 will be randomly allocated to sign up to the RHS Campaign for School Gardening and to receive the less intensive intervention and 16 schools will act as comparison schools. The schools will be randomly allocated to the associate (i.e. less intensive) intervention or the comparison group. The comparison schools will have no active RHS intervention during the trial. They will be offered delayed access to the less intensive intervention at the end of the study.

It will not be possible to blind schools to their intervention group because of the nature of the intervention. The fieldworkers will be blinded to the allocation of schools to the intervention (more or less intensive) and comparison arms of the study.

### Study population

#### Trial 1 inclusion criteria

All primary schools within the following London boroughs: Wandsworth; Tower Hamlets; Greenwich; and Sutton with classes in key stage 2 (years 3–6) will be invited to take part in the study.

#### Trial 2 inclusion criteria

All primary schools within the following London boroughs: Lewisham; Lambeth; Merton and Newham with classes in key stage 2 (years 3–6) will be invited to take part in the study.

### Exclusion criteria common to both trials

Independent schools, special schools and schools without all 4 year groups in key stage 2 at primary school (years 3–6) and small schools with less than 15 pupils/year group will be excluded.

### Proposed sample size

Cluster randomisation will be used, randomising at the school level, because the intervention will involve whole schools and participating classes. Based on results from our previous work on schools in a national sample, we estimate the standard deviation for the amount of vegetables eaten to be 85 g and for fruit 143 g. The associated intraclass correlation coefficient for total vegetables from Project Tomato was 12.5% and for fruit 11.4% (unpublished results). This sample of 50 children (one year 3 class and one year 4 class) from each school, will give a design effect of approximately 6.6 for vegetables and 7.1 for fruit to take account of the cluster randomisation. To have 90% power to detect a 0.5 portion difference in vegetable intake, 627 per group are required, i.e. about 13 schools using 2 classes from each school. To have 90% power to detect a 1 portion difference in fruit intake, 482 per group are required, i.e. about 10 schools. Based on results from our evaluation of the School Fruit and Vegetable Scheme, 75% who completed the Child And Diet Evaluation Tool (CADET) at baseline also completed the final follow up CADET. To allow for this margin of safety, 16 schools per group will be selected in each group apart from the intensive intervention group where it is only possible for 10 schools to be involved. The size of effect the study is powered to detect, (one half of a portion of vegetables or one portion of fruit) was chosen because it was considered the smallest improvement in intake that was worthwhile detecting with the achievable sample size, and considering the nature of the intervention.

### Discontinuation criteria

Analysis will follow the principle of intention-to-treat as far as possible. We will therefore include in analyses all schools and children initially randomised, including them for analysis purposes in the intervention group originally allocated to them. To this end, all reasonable and ethical steps will be taken to ensure completeness of follow-up of outcome measures.

### School withdrawal

If a school wishes to withdraw from the trial, the study team will post a data collection form to the head/class teacher along with a freepost envelope. The data collection form will record the following: reasons for withdrawal; whether anything could have been done to make taking part in the study easier; if they no longer want to take part in the intervention and receive information/training/materials and if they still allow us to use data collected to date and to collect data at round two i.e. follow-up collection in October 2011.

### Child withdrawal

A parent may request that an individual child is no longer part of the trial. This request may go either to the school, the RHS or the study team at the University of Leeds. Whoever is the first point of contact with the parent must inform the other relevant groups (school/RHS/University of Leeds) by telephone or letter; this will be recorded in the database. On receipt of this information the study team will send a letter to inform the class teacher that the child is to be withdrawn from the study. A data collection form and freepost envelope will be sent via the class teacher to the parent. A covering letter will make clear to the parent that while the child will not receive any questionnaire for completion, the child will not be left out of whole class activities as to do so would involve taking the child out of the class whilst these activities were occurring.

### Interim analysis and stopping rules

No interim analyses of trial outcomes are planned.

### Assessment of harm

On rare occasions, children or schools may need to discontinue the randomised intervention. This may, in most cases, be only a temporary withdrawal, for example, if a child injures themselves with a spade. Minor adverse reactions would not be grounds for discontinuing. However, these events will be captured by either the RHS advisor for the intensive interventions schools, or by the Nutritional Epidemiology Group team through the process measures email for the less intensive schools. All adverse events will be reported to the annual Trial Steering Committee meetings. However, the same procedures would apply as for school or individual withdrawal detailed above. Children who have been withdrawn from the trial due to an adverse reaction of some sort (eg. allergy etc.) will be followed up for 3 months by the study team after withdrawal to assess their condition.

### Randomisation

Cluster randomisation with school location and borough to identify each “cluster” will be used to randomise the schools. The schools will be randomised by their London borough location using Stata[39]. They will be randomly assigned, for trial one to the intense RHS intervention or the less intense RHS intervention; for trial two to the less intense intervention or the delayed intervention (comparison). If the schools had more than one Year 3 or Year 4 class, the same statistical method will be used to determine which class should be involved in the trial.

### Compliance with good practice

· All statistical analyses of primary and secondary trial outcomes will be carried out under the guidance of the trial statistician.

· CONSORT guidelines will be followed for presentation of results from cluster randomised trials [40]

· Presentation of results will conform to good practice for presentation of trials of complex interventions[41].

· The flow of both clusters and individuals through the trial, from assignment to analysis, will be presented using a flowchart, in accordance with CONSORT guidelines [40].

· Intraclass correlation coefficients from the multilevel analyses will be presented following good practice for cluster randomised trials.

### Ethical considerations

Ethical approval was obtained through the Leeds Institute of Health Sciences and Leeds Institute of Genetics, Health and Therapeutic joint ethics committee (Reference number: 09/012). Written informed consent from all schools will be obtained first and then obtained from all parents whose children are in the classes chosen to participate in the trial data collection. Schools and parents will be informed about the potential risks and benefits of participating in the trial through the information sheet. Participant’s parents will be given informed consent, with the opportunity to “opt-out” of the study if they did not wish their child to take part. If the parents wished their child not to participate in the study, they will still able to take part in the growing activities; however their food intake and child attitude and knowledge questionnaire will not be recorded.

### Data collection methods

Diet will be assessed using a validated questionnaire known as CADET (Child And Diet Evaluation Tool). CADET has been validated in an ethnically diverse population [42] and has been used to evaluate the ‘National Free School Fruit Scheme’ in primary school children [43,44] and has also been used in a large national randomised controlled trial of an intervention to maintain fruit and vegetable eating in Year 3 children once they are no longer eligible for free fruit [45]. Measures of socio-economic position are also included on the CADET. This includes a record of postcode, ethnic background and highest educational level of parents – these questions to be completed by the parent.

### Dietary assessment method: school and home food diaries

For this trial diet will be assessed using a modified version of the CADET questionnaire. To complete the School and Home Food diary participants tick each item consumed, under the appropriate meal time heading within the 24-hour period. The School Food Diary will be completed by an administrator at school for all school time meals, and then the children are given the Home Food Diary to take home and record for their evening snacks and meals, as well as breakfast the next day. The Home Food Diary is to be completed by a parent or carer. The following day the administrator will go back to the school to collect the Home Food Diary, and check that it has been completed accurately. If a child forgets to return their Home Food Diary a retrospective recall will be taken for all evening meals and breakfast by the administrator.

### Home food diary instruction DVD

To improve accuracy and completion of the Home Food Diary a short cartoon DVD was developed. Previously there were two pages of instructions for parents to read on how to complete the diary. The DVD was designed for children and parents to watch together before completing the Home Food Diary, with the aim of helping parents and children with low literacy ability or English as a second language to understand how to complete the diary.

### Knowledge and attitudes towards fruit and vegetables questionnaire

A short questionnaire to identify children’s knowledge and attitude towards fruit and vegetable consumption, and assess gardening activity levels will be administered in school. The knowledge questions assess children’s ability to recognise different fruit and vegetables. Children will be presented with a list of fruit and a list of vegetables (and a few herbs), with a colour picture for each, and they have to draw a line connecting the name with the right picture. The attitude questions are based on previously validated research by Somerset and Markwell, 2009 [46]. The gardening questions assess the children’s gardening experience; what they have grown and what they have tasted. This questionnaire will be read out to the children as a class, to help them with any difficult words. Children will complete the questionnaire individually.

### Process measures

Process evaluations are useful to identify adherence level to the intervention for each school. At baseline and follow-up, schools will be asked a set of gardening questions which are based on the RHS school bench marking system that ranks schools on their gardening activity levels from 1–5. An email consisting of different questions about gardening activities within the school, will be sent to the class teacher to capture what fruit and vegetables each school grows and harvests. This information will be captured via email in October 2010 for trial year one and October 2011 for trial year two. For the intense intervention trial schools in trial one, additional details regarding the intervention activities within each school will be captured by the regional advisor. For the less intense intervention, level of involvement in the twilight sessions for both trials one and two will be recorded. The regional advisor will keep a record of teacher’s attendance. The nature of this type of intervention allows schools to naturally tailor it to their individual needs. By monitoring what activities are undertaken in the school garden and in the classroom, there is an opportunity to explore how intervention elements might be associated with dietary change.

### Assessment and follow up

All measures will be taken at baseline and then at the end of the intervention, after two growing seasons e.g. within 15–17 months after baseline measurements were collected. Schools will have baseline measures taken when children are in the Spring Term of Year 3 and 4 (2010) and then again when these children are in Years 5 and 6, in the Autumn Term of that year (2011). The RHS Campaign will take place in schools over two growing seasons which will include the summers of 2010 and 2011. Support will be provided throughout the year to schools by the regional advisor.

### Study interventions

The RHS Campaign for School Gardening consists of two programmes. For the intensive intervention the schools known as Partner Schools receive the following:

· A day visit from the RHS regional advisor each half term to work in the garden with teachers and children (Summer Term 2010 to Summer Term 2011 inclusive).

· Follow up visits to aid lead teachers with planning (Autumn Term 2010 to Autumn Term 2011)

· General ongoing advice on the school garden, free seeds and tools

· 1 twilight teacher training session each term (Summer term 2010 to Summer term 2011 inclusive), based on seasonal tasks in the school garden (open to Partner School teachers and others from local schools)

· Free access to a wide range of teacher resources at http://www.rhs.org.uk/schoolgardening/

The role of the regional advisor is to assist the schools to develop a successful garden, through working directly with teachers and pupils to give them support and practical advice. They are also charged with helping schools overcome particular barriers to developing gardening within schools. Regional advisors have the expertise and experience to tie in gardening and growing activities with the National Curriculum and to run staff training sessions for teachers.

The less intensive intervention schools known as Associate Schools will work with the RHS by attending twilight training once a term at their nearby Partner school, to help support them in developing and using their school garden. Unlike the Partner Schools the Associate schools will not have direct support from the regional advisor. The regional advisor will be running these twilight sessions for them and provide the Associate Schools with advice as needed for their school garden.

Trial One consists of schools participating in both intervention groups mentioned above, whereas for Trial Two schools involved in the less intensive intervention/Associate Schools and a group of control schools are included. These control schools will not receive any support from the regional advisor during the period of the trial, they will however, be eligible for associate intervention at the end of the study. However, it is recognised that most schools will be engaging in some activity around this topic. Baseline evaluation of all schools will assess the level of active engagement with growing by these schools.

### Proposed outcome measures

#### The primary outcome measure will be the following

· Food:

· Daily portions of fruit and vegetable intake at follow-up (15 months after baseline)

· Daily portions of fruit intake

· Daily portions of vegetable intake

The effectiveness of either intervention would be determined by an increase in mean intake in one of the following: mean intake of fruit, mean intake of vegetables, or mean intake of fruit and vegetables at follow-up after adjusting for baseline

#### Secondary outcomes will be the following

· Nutrient:

· Total energy intake (MJ/day)

· Fat intake (g/day)

· Saturated fat (g/day)

· Salt intake (g/day)

· Sugars (g/day) including non milk extrinsic sugars

· Carotene intake (mg/day)

· Vitamin C intake (mg/day)

· Vitamin D intake (mg/day)

· Iron (μg/day)

· Fibre (g/day)

· Zinc (μg/day)

· Carbohydrates (g/day)

· Folate (μg/day)

· Foods:

· high in fat, salt or sugar and sugar sweetened beverages

· Behavioural:

· Children’s attitude to fruit and vegetable consumption

· School level:

· Involvement of schools in promoting consumption of fruit and vegetables (number of lessons devoted to school gardening and growing or learning about fruit and vegetables, school food policy, involvement in other national/local food related initiatives).

· Involvement by schools of parents in promoting consumption of fruit and vegetables among pupils.

· Process measures concerning the practicality of the intervention, timing, delivery, used and not used elements of the intervention.

### Statistical analysis

Baseline child-level variables between the two intervention groups will be assessed for the following variables:

School level:

· % children with English as an additional language

· % non-white children

· % children with free school meals eligibility

· % children defined as having special educational needs

Child level:

· Sex

· Age

· Each of the primary and secondary outcomes

### Primary analyses

A random or fixed intercepts model of primary and secondary outcomes will be used allowing for hierarchical structure of data caused by cluster randomisation: child within class within school. MLwiN [47] will be used for this analysis. The single covariate for the intervention group will be included in the model (treated as a random effect since schools in the trial are themselves a random sample from the population of schools).

### Secondary outcome analysis of the trial

To formally compare sub-groups, two predefined individual level variables (with interaction term) will be added one at a time, namely gender and ethnicity a p value of 0.01 will be used to take into account multiple testing. These will answer plausible questions, i.e. that the effect of the intervention differs by gender or by ethnicity. Unadjusted analyses originally performed in MLwiN will be repeated in Stata 10[39] using fixed effect Sandwich estimates to take account of the cluster randomisation to assess robustness of conclusions. These are exploratory analyses that are hypothesis generating.

### Multiple comparisons

No adjustment will be made for multiple comparisons of these pre-specified secondary analyses. All tests will use a 1% significance level, and report 95% confidence intervals.

### Subgroup analyses

The subgroup analyses outlined below will be investigated in an exploratory manner using the same modelling procedure as above. These will be tested at the 1% significance level.

· Differences between boys and girls

· Differences between school year groups

· Differences between quintiles of Index of Multiple Deprivation Scores

· Efficacy subset analysis

· Whilst it is often argued that the most meaningful results are based on Intention-to-Treat (ITT) principles, for intervention analysis such as this study, the degree to which schools participate in the intervention is vital to the main outcome. It would therefore be illuminating to compare the results from ITT and efficacy subset analysis to examine the differences in effect size generated [48].

### Knowledge and attitudes towards fruit and vegetables questionnaire

Multi-level regression analysis will be used to explore: fruit, vegetable, fruit and vegetable combined intake and their effects on children’s knowledge and attitude questions. These will be presented based on randomisation of children to the two trials.

### Process measures evaluation

The process measures questionnaire will explore the differences between schools with different levels of adherence to the intervention programmes. The process measures evaluation will also identify which schools have or have not improved their gardening curriculum from baseline to follow-up using regression analysis.

## Competing interest

The authors declare that they have no competing interests.

## Department of Health disclaimer

The views and opinions expressed therein are those of the authors and do not necessarily reflect those of the PHR programme, NIHR, NHS or the Department of Health.

## Authors’ contributions

JC conceived the study. CELE, JR, MC, and JC were applicants for the funding. MSC, CELE, and JC were involved in designing the study and drafting the protocol. All authors and the TSC read and approved the final protocol.

## Trial Steering Committee

Dr Cindy Cooper (Director, Sheffield Clinical Trials Research Unit Senior Research Fellow University of Sheffield) has agreed to act as chairperson. Graeme Slate (Learning Mentor – Forster Park Primary School) and Deirdre Walton (RHS Regions Manager) have agreed to act as independent members of the steering committee. The Trial Steering Committee will also act as the data monitoring committee.

## Pre-publication history

The pre-publication history for this paper can be accessed here:

http://www.biomedcentral.com/1471-2458/12/304/prepub
